# Serial neurocognitive changes following transcatheter aortic valve replacement: comparison between low and intermediate-high risk groups

**DOI:** 10.18632/aging.204202

**Published:** 2022-08-05

**Authors:** Tsung-Yu Ko, Hsien-Li Kao, Chih-Fan Yeh, Jiu-Hsiang Lin, Ching-Chang Huang, Ying-Hsien Chen, Chi-Chao Chao, Hung-Yuan Li, Chih-Yang Chan, Lung-Chun Lin, Yih-Sharng Chen, Ming-Jiuh Wang, Mao-Shin Lin

**Affiliations:** 1 Department of Internal Medicine, National Taiwan University Hospital, Taipei, Taiwan; 2 Graduate Institute of Clinical Medicine, Medical College, National Taiwan University, Taipei, Taiwan; 3 Institute of Statistical Science, Academia Sinica, Taipei, Taiwan; 4 Department of Neurology, National Taiwan University Hospital, Taipei, Taiwan; 5 Department of Surgery, National Taiwan University Hospital, Taipei, Taiwan; 6 Department of Anesthesiology, National Taiwan University Hospital, Taipei, Taiwan

**Keywords:** neurocognitive function, aortic stenosis, TAVR

## Abstract

Background: Data comparing the neurocognitive trajectory between low and intermediate-high risk patients following transcatheter aortic valve replacement (TAVR) is never reported.

Aims: To report serial neurocognitive changes up to 1 year post-TAVR in low and intermediate-high risk groups as well as overall cohort.

Methods: Prospective neurological assessments (NIHSS and Barthel Index), global cognitive tests (MMSE and Alzheimer Disease Assessment Scale–Cognitive Subtest, ADAS-cog) and executive performances (Color Trail Test A and B and verbal fluency), were applied at baseline, 3 months and 1 year post-TAVR.

Results: In overall cohort, persistent improvement to 1 year in MMSE, ADAS-cog, Color Trail Test A and B was found. According to the STS score, the study cohort was divided into low (<4%, *N* = 81) and intermediate-high (≧4%, *N* = 75) risk groups. The baseline neurologic and cognitive performance was significantly worse in intermediate-high risk group. Slight improvement on general neurological functions (Barthel index and proportion of NIHSS>0 patients) at 1 year could be observed only in intermediate-high risk group. In global cognitive assessments, improvement in MMSE and ADAS-cog at 1 year was found in both groups, but the proportion of cognitive improvement was more obvious in intermediate-high risk group. In Color Trail Tests and verbal fluency, significant and persistent improvement up to 1 year could be observed only in low risk group.

Conclusions: TAVR was associated with persistent improvement in global cognitive function, as well as in attention and psychomotor processing speed, up to 1 year in overall cohort. However, improvement in tests for executive functions can only be seen in low risk group.

## INTRODUCTION

Transcatheter aortic valve replacement (TAVR) has become an increasingly preferred alternative to surgical aortic valve replacement [1, 2], and its indication is rapidly expanding to population with low-to-intermediate surgical risk [3, 4]. Restoration of cardiac output after TAVR may lead to subsequent improvement in cerebral perfusion [5] and results in cognitive improvement. However, patients undergoing TAVR are at risk for early cerebrovascular events immediately after, or in the first few hours, following the procedure [6]. In addition, diffusion weighted magnetic resonance imaging (DWI) revealed new cerebral embolic lesions in up to 70% of patients after TAVR [7, 8]. In combination, these 2 mechanisms may negate cognition improvement [9, 10].

Previous reports on cognitive changes following TAVR gave variable results [11–16]. The majority of these reports’ sample sizes were relatively small and had only 2 cognitive assessments (pre- and 1–6 months post-TAVR), so the true longitudinal trajectory of post-TAVR cognition is thus lacking. In addition, all prior studies included only intermediate-high risk population. We hereafter present a prospective study with serial extensive neurocognitive function assessments done before, 3 months, and 1 year after TAVR, and compare the differences between low and intermediate-high risk groups.

## METHODS

### Patient population

**Figure 1:**
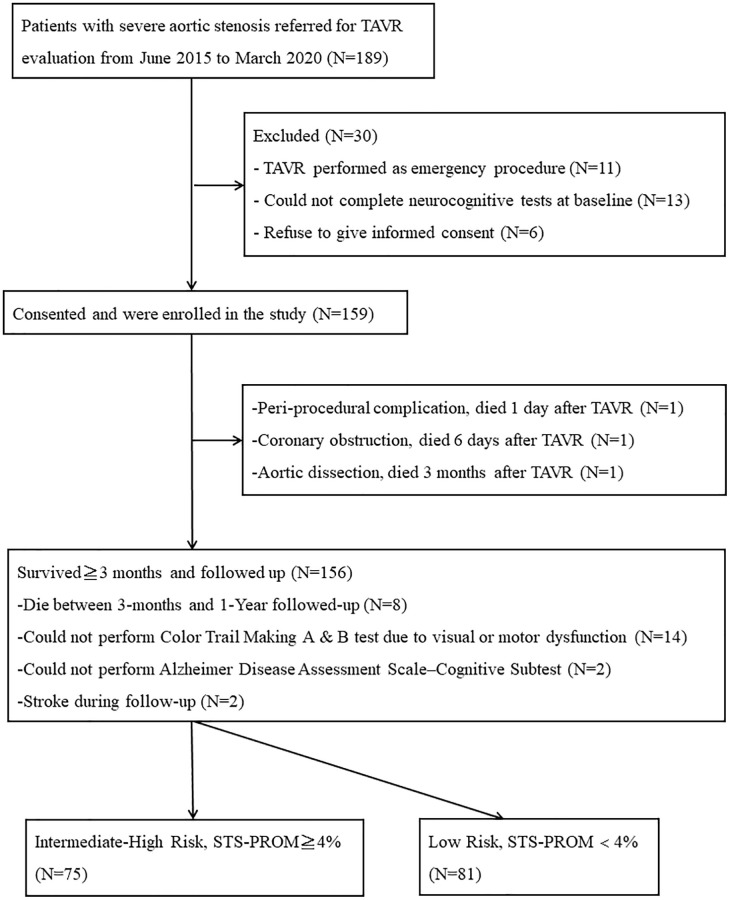
**Study flow chart outlining recruitment and grouping for patients depending on STS-PROM.** Abbreviations: TAVR: Transcatheter Aortic Valve Replacement; STS-PROM: Society of Thoracic Surgeons Predicted Risk of Mortality.

One hundred and eighty-nine consecutive patients with severe symptomatic aortic stenosis undergoing TAVR in National Taiwan University Hospital from June 2015 to March 2020 were screened for the study. The heart team determined TAVR indications, approach, and the type of transcatheter valves used. The self-expanding transcatheter valves (the CoreValve/Evolut R [Medtronic, Minneapolis, Minnesota], Portico [St. Jude Medical, Minneapolis, Minnesota]), the balloon-expandable transcatheter valves (the Sapien XT/Sapien 3 [Edwards Lifesciences, Irvine, California]), and other transcatheter valves (Lotus [Boston Scientific, Natick, Massachusetts]) were implanted. 33 patients were excluded from the study either because of TAVR performed as emergency procedure (*N* = 11), unable to complete neurocognitive tests at baseline (*N* = 13), refusal to give informed consent (*N* = 6), or death within 3 months following TAVR due to peri-procedural complications (*N* = 3). The final study population consisted of 156 patients (age: 80.2 ± 7.8 years; 56.4% women; Society of Thoracic Surgeons Predicted Risk of Mortality score, STS-PROM: 5.1 ± 3.9) who survived and completed neurocognitive assessment at least 3 months post-TAVR. 8 patients died between 3-months and 1-year post-TAVR, and another 2 patients suffered from new stroke during follow-up. These patients were, however, still included in the analysis (Figure 1).

All baseline, procedural, and follow-up data were collected prospectively in a dedicated database. The protocol was approved by the institutional ethics committee, and all patients provided written informed consent. Follow-up visits were scheduled at 3 months and 1 year post-TAVR. Definitions of procedural results were in accordance with the Valve Academic Research Consortium (VARC) consensus [17].

### Neurologic and cognitive assessment

All subjects underwent a standardized neurological and cognitive assessment performed by 2 trained, dedicated staffs, which were supervised by a neurologist, at baseline (within 1 week prior to the procedure), 3 months and 1 year post-TAVR. Neurological assessments included National Institutes of Health Stroke Scale (NIHSS) and Barthel index. The global cognitive assessment of global measures included the Mini-Mental State Examination (MMSE) [18, 19] and Alzheimer Disease Assessment Scale–Cognitive subscale (ADAS-Cog), a widely used rating instrument assessing memory, orientation, language, and ideational and constructional praxis [20, 21]. ADAS-Cog scores range from 0 to 70, with a higher score indicating lower performance. A cutoff of ≥3 points was used to define relevant change (deterioration or improvement) in MMSE and ADAS-cog. MMSE score < 26 points was considered cognitive impairment. [22].

Additional tests for executive functions, such as working memory, attention, and psychomotor processing speed, in patients with vascular-related cognitive impairment are also included. These included Color Trails Test A and B, and verbal fluency. The Color Trails Test was used to replace the more educational-dependent conventional Trail Making Test. It uses numbered colored circles with vivid pink or yellow backgrounds that are perceptible to colorblind individuals. For the Color Trails Test A, the respondent uses a pencil to rapidly connect circles numbered 1 through 25 in sequence. For the Color Trails Test B, the respondent rapidly connects numbered circles in sequence, but alternates between purple and yellow colors. The length of time to complete each test is recorded, along with qualitative features of performance indicative of brain dysfunction, such as near-misses, prompts, number sequence errors, and color sequence errors. The Color Trail Tests time scores are transformed to standard scores and T scores, and then categorized depending on age and educational level as the followings: 8, severely impaired; 7, moderately-to-severely impaired; 6, moderately impaired; 5, mildly-to-moderately impaired; 4, mildly impaired; 3, below average; 2, average; 1, above average [23–25]. The verbal fluency test consists of giving the respondent 60 seconds to verbally list as many things as possible in a specific category, such as fruits, vegetables, and fishes. It was assessed in letter and category fluency tasks, and performance on these tasks was related to indicators of vocabulary size, lexical access speed, updating, and inhibition ability [26].

In the 156 patients included in the study, 14 could not complete Color Trail Making A and B test due to color blindness or motor dysfunction. Of these, 5 were low-risk and 9 were intermediate-high risk patients. Another 2 patients were unable to perform ADAS-cog due to visual defect, of which 1 was low risk and 1 was intermediate-high patient (Figure 1).

### Statistical analysis

Quantitative variables are presented as mean ± standard deviation or median (interquartile range), depending on variable distribution. Normality was assessed using the Kolmogorov-Smirnov test. Categorical variables were compared using the chi-square test or Fisher exact test, whereas quantitative variables were compared with the use of the Student *t* test or Mann-Whitney *U* test depending on their distribution. Changes of cognitive function by time were analyzed by pair *t*-test or the Wilcoxon signed rank test depending on their distribution of baseline values. Changes of Color Trail Test A and B by time were analyzed by Mcnemar’s test. A 2-sided *P* value < 0.05 was considered statistically significant. Stata/SE 11.0 for Windows (StataCorp LP, TX, USA) was used for statistical analyses.

## RESULTS

### Patients and procedure characteristics

Baseline characteristics of excluded patients and study cohort were compared (Supplementary Table 1). There were no significant differences between these 2 populations, except for higher incidence of prior stroke or transient ischemic attack, higher proportion of patients with New York Heart Association functional class III/IV and higher surgical risk in excluded patients. According to the STS-PROM, the study cohort was divided into low (<4%, *N* = 81) and intermediate-high (≧4%, *N* = 75) risk groups. The baseline characteristics of patients in low and intermediate-high risk are shown in Supplementary Table 2. Although the clinical characteristics were significantly different between 2 groups, the baseline left ventricular function and the severity of aortic valve stenosis were comparable. The procedural characteristics and clinical outcomes in low and intermediate-high risk groups are shown in Supplementary Table 3. All of the patients in study cohort underwent TAVR through trans-femoral approach and under conscious sedation. None of patients received embolic protection device during procedure.

### Neurologic and cognitive assessment scores over time in overall cohort

Table 1 showed the changes of neurologic and cognitive assessments scores in whole study cohort. There was a trend of improved neurologic assessments over time, statistically significant in Barthel index score. In cognitive assessments, significant and persistent improvement in MMSE, ADAS-cog, Color Trails Test A and B were found. The proportion of cognitive impairment (defined as MMSE < 26) also significantly decreased over time.

**Table 1:** Serial changes of neurological and cognitive assessments in overall cohort.

	**Baseline evaluation *N* = 156**	**3 Month evaluation *N* = 156**	**1 Year evaluation *N* = 148**	**A vs. B *P* value**	**A vs. C *P* value**	**B vs. C *P* value**
	**A**	**B**	**C**
NIHSS						
Score	0 (0−0)	0 (0−0)	0 (0−0)	0.097	0.07	0.501
Number of score > 0	18 (11.5%)	12 (7.7%)	9 (6.1%)	0.286	0.057	0.581
Barthel index						
Score	100 (95−100)	100 (100−100)	100 (100−100)	0.019	0.0237	0.5504
Number of score < 100	40 (25.6%)	37 (23.7%)	28 (18.9%)	0.678	0.087	0.189
MMSE						
Score	27 (22−29)	28 (25−30)	29 (25−30)	0.0014	0.001	0.282
Number of score < 26	61 (39.1%)	49 (31.4%)	41 (27.7%)	0.029	0.0009	0.524
ADAS-cog	4 (1−10)	2 (1−6)	2 (0−5)	<0.0001	<0.0001	0.333
Color Trail Test A (category)	8 (4−8)	7 (3−8)	7 (3−8)	0.0187	0.0424	0.601
Color Trail Test B (category)	8 (6−8)	8 (4−8)	8 (3−8)	0.0126	0.0002	0.0438
Verbal fluency	27.7 ± 9.5	28.7 ± 9.1	28.3 ± 10.0	0.0375	0.3388	0.3544

Values are number (%), median (interquartile range) or mean (standard deviation) if normally distributed. Abbreviations: NIHSS: National Institutes of Health Stoke Scale; ADAS: Alzheimer Disease Assessment Scale; MMSE: Mini-Mental State Examination score.

### Baseline neurologic and cognitive assessments between low and intermediate-high risk groups

The comparison of baseline neurologic and cognitive functions between low and intermediate-high groups was shown in Supplementary Table 4. Both neurologic and cognitive performances were significantly worse in intermediate-high risk group, except for Color Trail Test B. The proportion of neurologic dysfunction (NIHSS > 0, Barthel index < 100) and cognitive impairment (MMSE < 26) were also significantly higher in intermediate-high risk group.

### Neurologic assessment scores over time by groups

In intermediate-high risk group, the numbers of patients with NIHSS > 0 decreased at 1 year as compared to baseline. Barthel index increased numerically over time, but the numbers and proportion of patients with Barthel index < 100 were not statistically different. In low risk group, there was no significant change in NIHSS and Barthel index over time, due to low proportion of neurologic dysfunction at baseline (Table 2). Supplementary Figure 1A and 1B show the percentage of patients with changes in NIHSS. In intermediate-high risk group, neurologic deterioration (defined as score increase) was observed in 6 patients (6/75, 8%) at 3 months and 4 patients (4/69, 5.8%) at 1 year, whereas neurologic improvement (defined as score decrease) in 14 patients (14/75, 18.7%) at 3 months and 10 patients (10/69, 14.5%) at 1 year. The proportion of deterioration and improvement in low risk group were significantly different from the intermediate-high risk group, with much smaller deterioration and improvement at 3 months and at 1 year. The percentage of patients with deterioration (defined as score decrease) or improvement (defined as score increase) in Barthel index (Supplementary Figure 1C and 1D) shows comparable patterns with those in NIHSS.

**Table 2:** Serial changes of neurological and cognitive assessments in low and intermediate-high risk groups.

	**Baseline Evaluation**	**3 Month Evaluation**	**1 Year Evaluation**	**A vs. B *P* value**	**A vs. C *P* value**	**B vs. C *P* value**
	**A**	**B**	**C**
Intermediate-High Risk	*N* = 75	*N* = 75	*N* = 69			
NIHSS						
Score	0 (0−0)	0 (0−0)	0 (0−0)	0.073	0.103	0.518
Number of score > 0	16 (21.3%)	8 (10.7%)	7 (10.1%)	0.077	0.022	1
Barthel index						
Score	100 (85−100)	100 (90−100)	100 (95−100)	0.088	0.039	0.735
Number of score < 100	28 (37.3%)	25 (33.3%)	20 (29.0%)	0.581	0.21	0.607
MMSE						
Score	25 (22−29)	27 (23−29)	27 (23−29)	0.0002	0.0017	0.554
Number of score < 26	39 (52.0%)	27 (36.0%)	27 (39.1%)	0.008	0.006	0.754
ADAS-cog	7 (3−11)	4 (1−9)	3 (1−7)	<0.0001	<0.0001	0.459
Color Trail Test A (category)	8 (6−8)	8 (3−8)	8 (6−8)	0.054	0.572	0.0045
Color Trail Test B (category)	8 (8−8)	8 (8−8)	8 (8−8)	0.751	0.141	0.519
Verbal fluency	24.9 ± 9.9	25.2 ± 8.5	24.1 ± 8.6	0.57	0.263	0.2255
Low Risk	*N* = 81	*N* = 81	*N* = 79			
NIHSS						
Score	0 (0−0)	0 (0−0)	0 (0−0)	0.429	0.989	0.317
Number of score > 0	2(2.5)	4(4.9)	4 (5.1)	0.414	0.414	0.987
Barthel index						
Score	100 (100−100)	100(100-100)	100 (100−100)	0.97	0.4	0.238
Number of score < 100	12 (14.8)	12 (14.8)	8 (10.1)	1	0.248	0.103
MMSE						
Score	29 (25−30)	29 (25−30)	29 (26−30)	0.398	0.013	0.017
Number of score < 26	22 (27.2%)	22 (27.2%)	14 (17.7%)	1	0.109	0.146
ADAS-cog	1 (0−7)	1 (0−4)	1 (0−3)	0.0004	0.005	0.203
Color Trail Test A (category)	6 (3−8)	6 (2−8)	5 (2−8)	0.074	0.0013	0.0259
Color Trail Test B (category)	8 (4−8)	8 (3−8)	5 (2−8)	0.0017	<0.0001	0.0037
Verbal fluency	30.4 ± 8.2	31.9 ± 8.5	32.0 ± 9.7	0.0261	0.054	0.897

Values are number (%), median (interquartile range) or mean (standard deviation) if normally distributed. Abbreviations: NIHSS: National Institutes of Health Stoke Scale; ADAS: Alzheimer Disease Assessment Scale; MMSE: Mini-Mental State Examination score.

### Global cognitive assessment scores over time by groups

**Figure 2:**
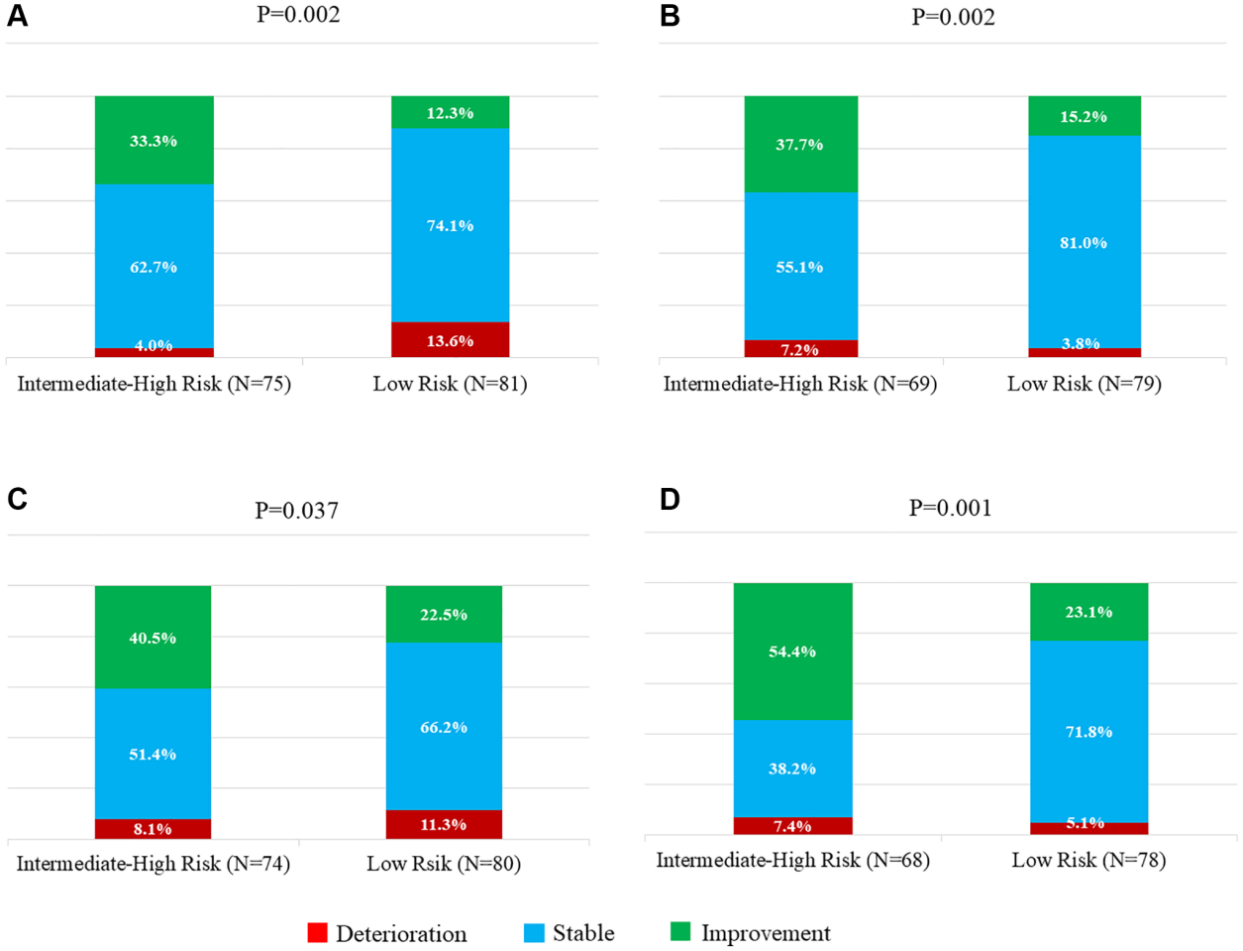
**Percentage of patients with changes in MMSE and ADAS-cog.** Deterioration or improvement was defined as change of ≥3 points decrease or increase in the MMSE score. (**A**) Baseline to 3 months. (**B**) Baseline to 1 year. Deterioration or improvement was defined as change of ≥3 points increase or decrease in the ADAS-cog score. (**C**) Baseline to 3 months. (**D**) Baseline to 1 year. Abbreviations: MMSE: Mini-Mental State Examination score; ADAS-cog: Alzheimer Disease Assessment Scale–Cognitive Subtest.

Persistent improvement in MMSE scores, as well as decrease in proportion of MMSE < 26, could be observed in intermediate-high risk group. But in low risk group, MMSE scores remained unchanged at 3 months, but showed significant improvement at 1 year as compared to baseline. Both groups showed significant improvement in ADAS-cog scores at 3 months, and persistent up to 1 year (Table 2). Figure 2A and 2B show the percentage of patients with changes in MMSE. In intermediate-high risk group, cognitive deterioration (defined as ≥3 points decrease) was observed in 3 patients (3/75, 4.0%) at 3 months and 5 patients (5/69, 7.2%) at 1 year, whereas cognitive improvement (defined as ≥3 points increase) in 25 patients (25/75, 33.3%) at 3 months and also 26 patients (26/69, 37.7%) at 1 year. The proportion of deterioration and improvement in low risk group were significantly different from the intermediate-high risk group, with much smaller improvement at 3 months (10/81, 12.3%) and at 1 year (12/79, 15.2%). Figure 2C and 2D present the percentage of patients with changes in ADAS-cog, demonstrating comparable patterns with those in MMSE. Much higher percentages of cognitive improvement (defined as ≥3 points decrease) could be observed at 3 months (30/74, 40.5%) and 1 year (37/68, 54.4%) in intermediate-high risk group, than those in low risk group.

### Neurocognitive tests for executive functions over time by groups

**Figure 3:**
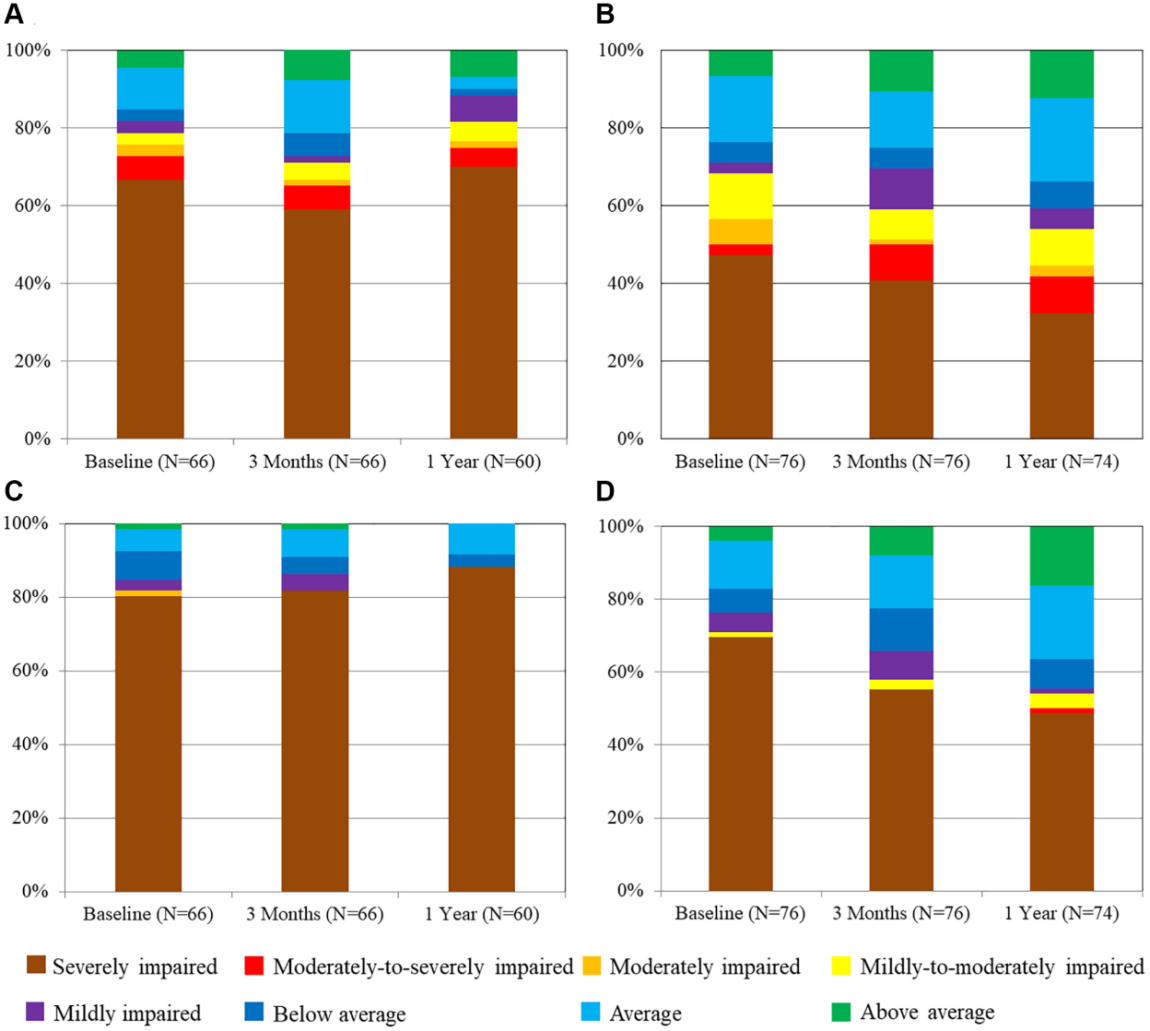
**Evolution of the percentage of cognitive category in color trails test A and B over time.** (**A**) Color Tails Test A in intermediate-high risk group. (**B**) Color Tails Test A in low-risk group. (**C**) Color Tails Test B in intermediate-high risk group. (**D**) Color Tails Test B in low-risk group.

**Figure 4:**
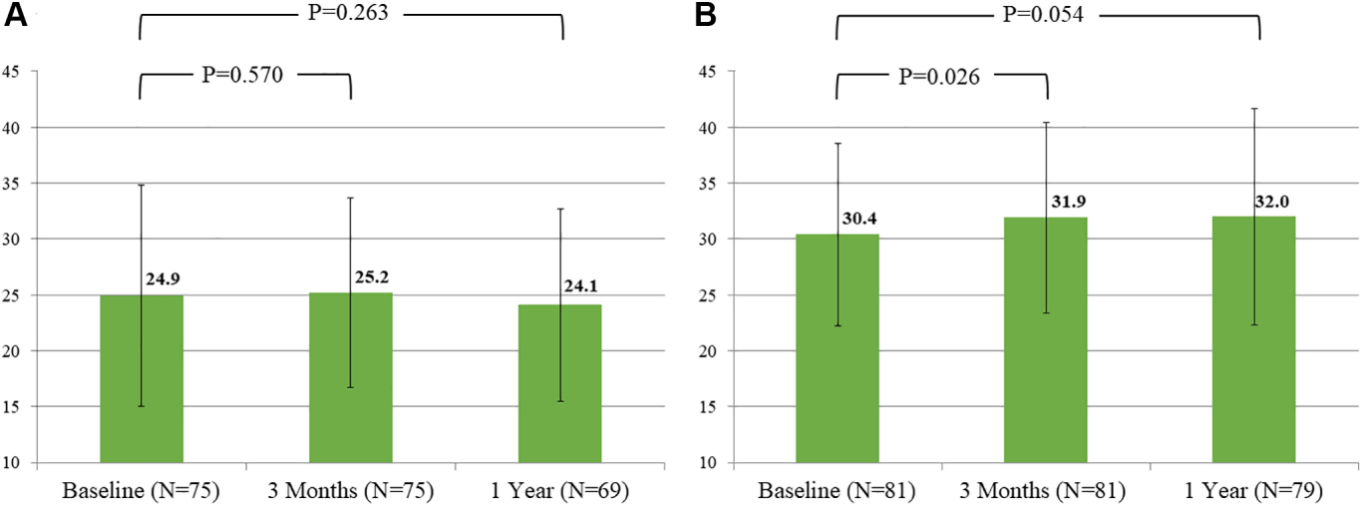
**Evolution of mean score of verbal fluency over time by groups.** (**A**) Intermediate-high risk group. (**B**) Low risk group.

Figure 3 showed the changes of the percentage of executive cognitive category in Color Trails Test A and B over time. Improvement of both tests at 3 months and persistent at 1 year could be observed only in low risk group, but not in intermediate-high risk group (Table 2). Similar results were also found in Verbal Fluency (Figure 4), which showed a trend of improvement only in low risk group.

## DISCUSSION

The present study reports the mid- to long-term neurologic and cognitive trajectory in TAVR recipients using an extensive battery of neurologic and cognitive tests. It is the first to compare cognitive changes between low and intermediate-high risk populations. The main findings of the present study are: 1) TAVR was associated with improvement in global cognitive function as well as attention, psychomotor processing speed, and persistent up to 1 year in overall cohort. 2) Numerical improvement in global cognitive assessments (MMSE and ADAS-cog) at 1 year could be observed in both groups, but the proportion of improvement is lower in low risk group than those in intermediate-high risk group. 3) In cognitive assessments examining attention, psychomotor processing speed, and executive function, improvement could only be observed in low-risk group at 3 months and persistent up to 1 year. 4) The trend of neurologic improvement in Barthel index and NIHSS found in intermediate-high risk group was not seen in low risk group.

Individuals with severe aortic stenosis are frequently accompanied with multiple co-morbidities and high atherosclerotic burden and at risk of vascular cognitive impairment [27, 28]. Cerebral hypoperfusion due to reduced cardiac output or cerebral arterial stenosis plays a major role on cognitive decline [29, 30]. Previous reports have shown improvement in neurocognitive functions following successful stenting in patient with carotid stenosis and abnormal cerebral perfusion, using the same battery of tests as the present study [31–33]. An association between increase in cardiac output and increase in cerebral blood flow in 31 patients following TAVR was found by Vlastra et al. [5]. Schoenenberger et al. reported cognitive improvement in patients with pre-TAVR aortic valve area less than 0.6 cm^2^ [12]. Auffret et al. also reported improvement in mean Montreal Cognitive Assessment 30-day and 1-year post-TAVR in 51 patients [11]. These findings all suggested the potential beneficial effect of augmented cerebral perfusion on cognition post TAVR.

However, this positive effect may be negated by the inherent risk of early cerebrovascular events and procedural embolization. In fact, post-TAVR cognitive decline has been reported [16]. Obviously, major neurological complication will harm patient. There were 2 major neurological complications occurred in our present study. In fact, these parameters in the intermediate-high risk patients even saw improvement after TAVR, reflecting improved general performance in these groups of fragile patients. Silent new cerebral DWI-lesions, consequent of small debris embolization, has been well documented after TAVR. Albeit neurologically silent, they may contribute to the cognitive decline early after TAVR [9, 10]. The effect may be transient, and insignificant at 1 year in intermediate risk patients [34, 35]. But as TAVR indication is expanding to lower risk and younger patients, it seems mandatory now to investigate the net impact of TAVR on long-term cognitive function with appropriate tests.

Different tests were designed to evaluate different aspects of cognitive function, and their results in different population may also be different. MMSE and ADAS-Cog are the commonly applied tools to assess global cognitive function, but they can only assess relatively stable aspects of cognition in the population of mild-to-moderate dementia [33]. In low surgical risk patients with relatively good cognitive performance, both tests may exhibit “ceiling effect” and overall beneficial effect of TAVR may be easily masked. On the other hand, complex executive tests such as Color Trails Test A and B and verbal fluency are suitable to detect subtle cognitive changes following TAVR in patients with good baseline cognitive performance, but insensitive for intermediate-high risk patients who already exhibit baseline permanent dysfunction. This “floor effect” had been reported to obscure changes in patients whose executive function were already permanently impaired [27, 28, 36], and explained why prior studies failed to demonstrate any significant changes in cognitive performance after TAVR [11, 37–39]. The observation of our study also verifies above points of view. As compared to intermediate-high risk group, the proportion of global cognitive improvement at 1 year was obviously lower in low risk group. The results are mostly due to lower portion of baseline cognitive impairment in low risk group and ceiling effect of MMSE and ADAS-cog. On the contrary, the improvement of high executive function was found only in low risk group rather than intermediate-high risk group, which had very high proportion of patients with severely impaired cognition at baseline.

The “practice effect” in repeating executive cognitive evaluation within a short interval may raise concerns in the interpretation of cognitive improvement [40, 41]. Unless a “sham” control is incorporated, this effect is difficult be clarified. In a study enrolling 36 healthy adults averaging 47 years-old, strong practice effects occurs in the initial 3-month phase of high-frequency repetitive cognitive testing. After 3 months and upon reduced testing frequency, a stabilization/plateau of the cognitive level until 1 year was observed [42]. However, such practice effects may be age-specific. Mitrushina et al. explored the magnitude of practice effect in repeated administration of different cognitive domains in 122 normal elderly subjects between ages 57 and 85 [43]. Improved cognitive performance in retest was likely to occur in younger people due to practice effect, but an opposite pattern was seen in individuals above 75 years-old, in whom a cognitive decline from test to retest was demonstrated. In our study, the average age of overall cohort was 80.2 and of low risk group was 77.6, and the tests were separated at least 3 months apart, so practice effect is less likely to occur.

### Study limitations

The present single center study has several limitations. Around 16% of patients were excluded because of critical condition or incapability to complete neurocognitive testing at baseline. The excluded patients were mostly at intermediate-high surgical risk, and more likely to experience cognitive changes post-TAVR. Thus, a potential underestimation of the true incidence of both cognitive improvement and deterioration could not be excluded. 8 patients died between 3 months and 1 year post-TAVR. This may also deliver a potential bias in the 1-year cognitive performance, as the number of participants with cognitive deterioration after TAVR may have been underrepresented. Brain magnetic resonance imaging were not applied and new cerebral DWI lesions were not examined. Future studies may be needed to establish the impact of “silent” embolism on cognition, and the quantitative relationship of cognitive changes to the extent of embolization. The effects of operator experience and device evolution within the study period cannot be controlled. Multicenter registry with larger patient number treated with contemporary techniques and devices over a shorter enrollment period are mandatory.

## CONCLUSIONS

TAVR was associated with improvement in global and executive cognitive functions, at 3 months post-TAVR and persistent up to 1 year. Global cognitive changes could be detected more in intermediate-high risk group, while the executive tests revealed more cognitive improvement in low risk group.

## Supplementary Materials

Supplementary Figure 1

Supplementary Tables
