# Supplementary material for: Serial neurocognitive changes following transcatheter aortic valve replacement: comparison between low and intermediate-high risk groups
Source: Aging (Albany NY). 2022 Aug 5;14(15):6111–27. doi: 10.18632/aging.204202 (PMC9417238; doi:10.18632/aging.204202)
Supplement: Supplementary Figure 1 [file aging-14-204202-s001.pdf]

SUPPLEMENTARY FIGURE

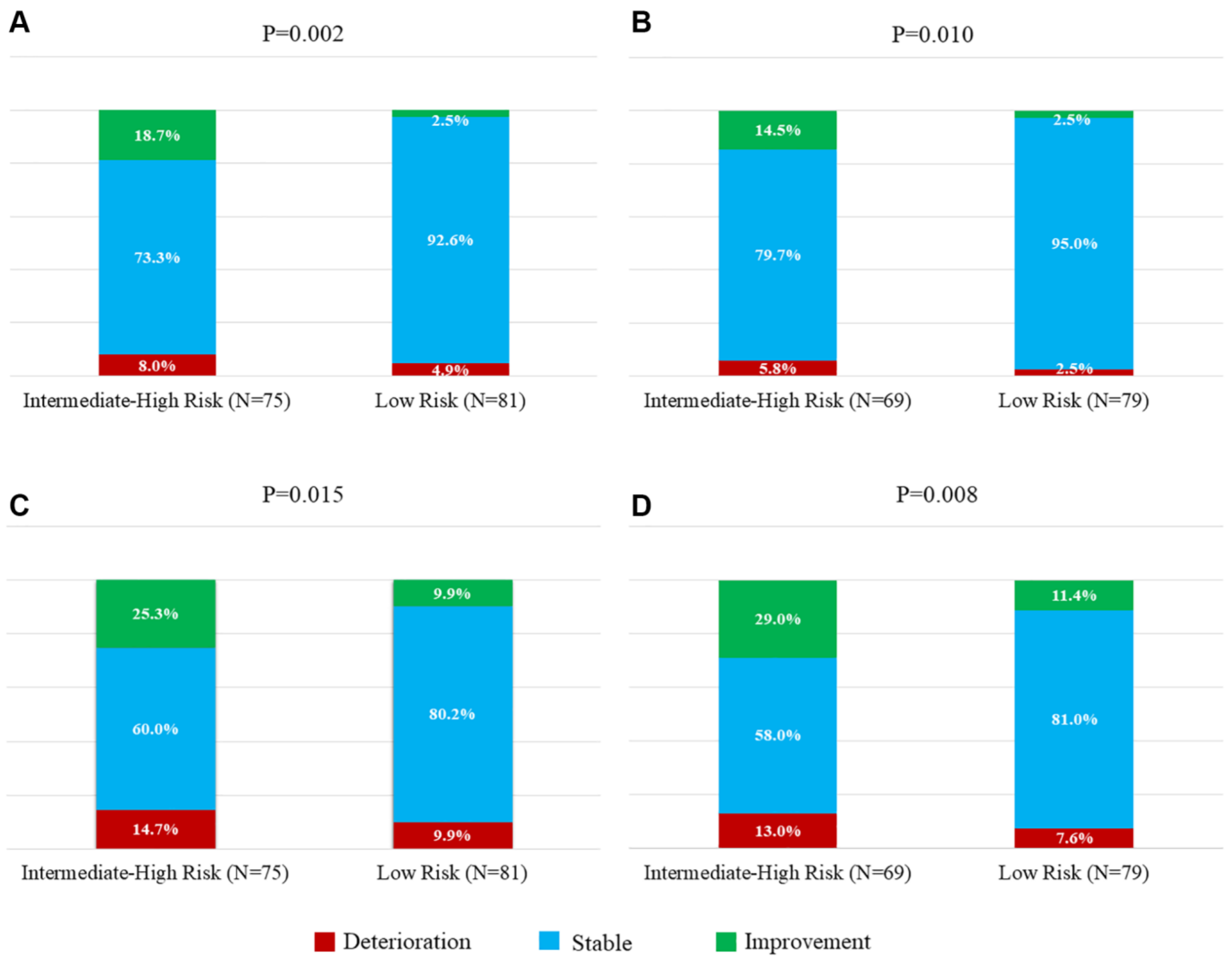

Supplementary Figure 1. Percentage of patients with changes in NIHSS and Barthel index. Deterioration or improvement was defined as score increase or decrease in the NIHSS. (**A**) Baseline to 3 months. (**B**) Baseline to 1 year. Deterioration or improvement was defined as score decrease or increase in the Barthel index. (**C**) Baseline to 3 months. (**D**) Baseline to 1 year. Abbreviation: NIHSS: National Institutes of Health Stoke Scale.
