# Supplementary material for: Serial neurocognitive changes following transcatheter aortic valve replacement: comparison between low and intermediate-high risk groups
Source: Aging (Albany NY). 2022 Aug 5;14(15):6111–27. doi: 10.18632/aging.204202 (PMC9417238; doi:10.18632/aging.204202)
Supplement: Supplementary Tables [file aging-14-204202-s002.pdf]

## SUPPLEMENTARY TABLES

**Supplementary Table 1. Comparison of baseline characteristics between excluded and included patients.**

| | Excluded patients (*N* = 33) | Study cohort (*N* = 156) | *P* value |
|---|---|---|---|
| Female sex | 21 (63.6%) | 88 (56.4%) | 0.445 |
| Age (year) | 82.3 ± 8.8 | 80.2 ± 7.8 | 0.169 |
| Body mass index, kg/m2 | 23.6 ± 4.6 | 24.3 ± 4.3 | 0.416 |
| Hypertension | 24 (72.7%) | 99 (63.5%) | 0.310 |
| Diabetes mellitus | 13 (39.4%) | 55 (35.3%) | 0.653 |
| Hyperlipidemia | 9 (27.3%) | 41 (26.3%) | 0.907 |
| Coronary artery disease | 16 (48.5%) | 63 (40.4%) | 0.391 |
| Peripheral artery disease | 7 (21.2%) | 19 (12.2%) | 0.171 |
| Prior myocardial infarction | 3 (9.1%) | 4 (2.6%) | 0.103 |
| Prior stroke or transient ischemic attack | 6 (18.2%) | 10 (6.4%) | 0.039 |
| Chronic kidney disease | 14 (42.4%) | 47 (30.1%) | 0.170 |
| Chronic lung disease | 1 (3.0%) | 12 (7.7%) | 0.336 |
| Permanent pacemaker | 1 (3.0%) | 7 (4.5%) | 1.000 |
| EuroSCORE II, % | 10.4 ± 10.1 | 3.9 ± 3.2 | <0.0001 |
| STS-PROM, % | 7.8 ± 4.3 | 5.1 ± 3.9 | 0.0006 |
| NYHA Fc 3/4 | 33 (100%) | 121 (77.6%) | 0.001 |
| Echocardiography | | | |
| LVEF, % | 65.2 ± 14.1 | 65.0 ± 13.0 | 0.939 |
| Aortic valve area, $cm^2$ | 0.76 ± 0.21 | 0.77 ± 0.18 | 0.730 |
| Mean pressure gradient, mmHg | 42.2 ± 22.6 | 42.6 ± 17.6 | 0.918 |

Values are *n* (%) or mean ± SD. Abbreviations: Euroscore: European System for Cardiac Operative Risk Evaluation; STS-PROM: Society of Thoracic Surgeons Predicted Risk of Mortality; NYHA Fc: New York Heart Association functional class; LVEF: left ventricular ejection fraction.

**Supplementary Table 2. Baseline characteristics in intermediate-high and low risk group.**

| | Intermediate-high risk (*N* = 75) | Low risk (*N* = 81) | *P* value |
|---|---|---|---|
| Female sex | 46 (61.3%) | 42 (51.9%) | 0.233 |
| Age (year) | 82.9 ± 6.8 | 77.6 ± 7.8 | <0.0001 |
| Body mass index, kg/m2 | 23.3 ± 3.9 | 25.3 ± 4.4 | 0.0035 |
| Hypertension | 48 (64.0%) | 51 (62.9%) | 0.893 |
| Diabetes mellitus | 34 (45.3%) | 21 (25.9%) | 0.011 |
| Hyperlipidemia | 17 (22.7%) | 24 (29.6%) | 0.324 |
| Coronary artery disease | 37 (49.3%) | 26 (32.1%) | 0.028 |
| Peripheral artery disease | 14 (18.7%) | 5 (6.2%) | 0.017 |
| Prior myocardial infarction | 3 (4.0%) | 1 (1.2%) | 0.352 |
| Prior stroke or transient ischemic attack | 7 (9.3%) | 3 (3.7%) | 0.352 |
| Chronic kidney disease | 37 (49.3%) | 10 (12.4%) | <0.0001 |
| Chronic lung disease | 5 (6.7%) | 7 (8.6%) | 0.644 |

| | | | |
|---|---|---|---|
| Permanent pacemaker | 4 (5.3%) | 3 (3.7%) | 0.711 |
| EuroSCORE II, % | 5.6 ± 3.8 | 2.4 ± 1.1 | <0.0001 |
| STS-PROM, % | 8.1 ± 3.8 | 2.4 ± 0.8 | <0.0001 |
| NYHA Fc 3/4 | 70 (93.3%) | 51 (63.0%) | <0.0001 |
| Echocardiography | | | |
| LVEF, % | 63.3 ± 15.1 | 66.7 ± 10.4 | 0.126 |
| Aortic valve area, $cm^2$ | 0.75 ± 0.20 | 0.79 ± 0.15 | 0.176 |
| Mean pressure gradient, mmHg | 42.9 ± 19.6 | 42.2 ± 15.6 | 0.821 |

Values are *n* (%) or mean ± SD. Abbreviations: Euroscore: European System for Cardiac Operative Risk Evaluation; STS-PROM: Society of Thoracic Surgeons Predicted Risk of Mortality; NYHA Fc: New York Heart Association functional class; LVEF: left ventricular ejection fraction.

**Supplementary Table 3. Comparison of procedural characteristics and clinical outcomes between low and intermediate-high risk groups.**

| | **Intermediate-high risk (*N* = 75)** | **Low risk (*N* = 81)** | ***P* Value** |
|---|---|---|---|
| Trans-femoral Approach | 75 (100.0%) | 81 (100.0%) | 1.000 |
| Valve Type | | | 0.059 |
| CoreValve/ Evolute R | 39 (52.0%) | 29 (35.8%) | |
| Sapien XT/ Sapien 3 | 29 (38.7%) | 46 (56.8%) | |
| Lotus | 6 (8.0%) | 3 (3.7%) | |
| Portico | 1 (1.3%) | 3 (3.7%) | |
| Need for 2nd Valve | 1 (1.3%) | 2 (2.5%) | 1.000 |
| Total contrast medium used, ml | 170.8 ± 50.5 | 168.3 ± 65.7 | 0.801 |
| Total procedural time, min | 102.6 ± 35.8 | 93.9 ± 37.8 | 0.146 |
| In-hospital outcome | | | |
| Acute kidney injury | 3 (5.4%) | 4 (4.9%) | 1.000 |
| New LBBB | 29 (38.7%) | 12 (14.8%) | 0.001 |
| New permanent pacemaker | 11 (14.7%) | 3 (3.7%) | 0.023 |
| Vascular complication, major | 3 (4.0%) | 1 (1.2%) | 0.358 |
| 1 Year Clinical outcome, | | | |
| Death | 6 (8.0%) | 2 (2.5%) | 0.155 |
| Cardiovascular Death | 1 (1.3%) | 0 (0.0%) | 0.481 |
| Rehospitalization | 27 (36.0%) | 16 (19.8%) | 0.124 |
| Heart failure rehospitalization | 8 (10.7%) | 6 (7.4%) | 0.477 |
| Stroke or transient ischemic attack | 2 (2.7%) | 0 (0.0%) | 0.230 |

Values are *n* (%), mean ± SD or median (interquartile range). Abbreviation: LBBB: left bundle branch block.

**Supplementary Table 4. Comparison of baseline neurologic and cognitive assessments between low and intermediate-high risk groups.**

| | Intermediate-high risk (*N* = 75) | Low risk (*N* = 81) | *P* Value |
|---|---|---|---|
| NIHSS | | | |
| Score | 0 (0–0) | 0 (0–0) | 0.001 |
| Number of score > 0 | 16 (21.3%) | 2 (2.5%) | <0.0001 |
| Barthel index | | | |
| Score | 100 (85–100) | 100 (100–100) | 0.0012 |
| Number of score < 100 | 28 (37.3%) | 12 (14.8%) | 0.001 |
| MMSE | | | |
| Score | 25 (22–29) | 29 (25–30) | <0.0001 |
| Number of score < 26 | 39 (52.0%) | 22 (27.2%) | 0.001 |
| ADAS-cog | 7 (3–11) | 1 (0–7) | <0.0001 |
| Color Trail Test A (category) | 8 (6–8) | 6 (3–8) | 0.023 |
| Color Trail Test B (category) | 8 (8–8) | 8 (4–8) | 0.121 |
| Verbal fluency | 24.9 ± 9.9 | 30.4 ± 8.2 | 0.0002 |

Values are number (%), median (interquartile range) or mean (standard deviation) if normally distributed. Abbreviations: NIHSS: National Institutes of Health Stoke Scale; ADAS-cog: Alzheimer Disease Assessment Scale–Cognitive Subtest; MMSE: Mini-Mental State Examination score.
